# Supplementary material for: FastqPuri: high-performance preprocessing of RNA-seq data
Source: BMC Bioinformatics. 2019 May 3;20:226. doi: 10.1186/s12859-019-2799-0 (PMC6500068; doi:10.1186/s12859-019-2799-0)
Supplement: Supplementary file 2 — Archive of FastqPuri. Archive containing all files needed to install and run FastqPuri v1.0.6. Date stamp March 22, 2019. (GZ 47,819 kb) [file 12859_2019_2799_MOESM2_ESM.gz › FastqPuri-1.0.6/html/md_README_Qreport.html]

My Project: QReport user manual


My Project

QReport user manual

Obtains statistics from the reads quality of a fastq file. The binary output is then read by an `Rmd` script that generates a html visualization of the data.

## Running the program

Usage `C` executable (in folder `bin`):

Usage: ./Qreport -i <INPUT\_FILE.fq> -l <READ\_LENGTH>

-o <OUTPUT\_FILE> -t [NUMBER\_OF\_TILES] -q [MINQ]

-n [#\_QUALITY\_VALUES] -f [FILTER\_STATUS]

Reads in a fq file (gz, bz2, z formats also accepted) and creates a

quality report (html file) along with the necessary data to create it

stored in binary format.

Options:

-v prints package version.

-h prints help dialog.

-i Input file [\*fq|\*fq.gz|\*fq.bz2]. Mandatory option.

-l Read length. Length of the reads. Mandatory option.

-o Output file prefix (with NO extension). Mandatory option.

-t Number of tiles. Optional (default 96).

-q Minimum quality allowed. Optional (default 27).

-n Number of different quality values allowed. Optional (default 46).

-f Filter status: 0 original file, 1 file filtered with trimFilter,

2 file filtered with another tool. Optional (default 0).

## Output description

- Binary output:
  - `int` (4B) : read length (`read_len`),
  - `int` (4B) : number of tiles (`ntiles`),
  - `int` (4B) : minimum quality accepted (`minQ`),   
    \* `int` (4B) : number of possible qualities (`nQ`),
  - `int` (4B) : number of reads (`nreads`),
  - `int` (4B) : number of reads containing N's (`reads_wN`),
  - `int` (4B) : size of `lowQ_ACGT_tile`, see below (`sz_lowQ_ACGT_tile`),
  - `int` (4B) : size of `ACGT_tile`, see below (`sz_ACGT_tile`),
  - `int` (4B) : size of `reads_MlowQ`, see below (`sz_reads_MlowQ`),
  - `int` (4B) : size of `QPosTile_table`, see below (`sz_QPosTile_table`),
  - `int` (4B) : size of `ACGT_pos`, see below (`sz_ACGT_pos`),
  - `ntiles*int` (4x`ntiles`B) : tile tags (`tile_tags`),
  - `ntiles*int` (4x`ntiles`B) : lane tags (`lane_tags`),
  - `nQxint` (4x`nQ`B) : quality tags (`quality tags`),
  - `5 x ntiles x (long int)` (5x`ntiles`x8B) : # (A,C,G,T,N) per tile with low quality (`lowQ_ACGT_tile`),
  - `5 x ntiles x (long int)` (5x`ntiles`x8B) : # (A,C,G,T,N) per tile (`ACGT_tile`),
  - `(read_len+1) x (long int)` ((`read_len`+1)x8B) : # reads with at least `m` low quality base callings (`reads_MlowQ`),
  - `ntiles x read_len x nQ x (long int)` (`ntiles`x`read_len`x`nQ`x8B) : base callings per tile per position with a given quality (`QposTile_table`).
  - `5 x read_len x (long int)` (5x`read_len`x8B ): # (A,C,G,T,N) per position, (`ACGT_pos`)
- html output:
  - Table with general information,
  - Plot 1: per base sequence quality,
  - Plot 2: # reads with at least m low Q base callings,
  - Plot 3: Low Q base calling proportion per nucleotide type per tile per lane,
  - Plot 4: Average quality per position per tile per lane,
  - Plot 5: Low Q base calling proportion per position per tile per lane,
  - Plot 6: Low Q base calling proportion per position for all tiles.
  - Plot 7: Nucleotide content per position

**NOTE** If the data were sequenced on more than one lane, in Plots 3, 4 and 5 there will be a heatmap pro lane.

## Example

An example is given in the folder `examples/QReport_Sreport`. To run an example, type,

$ cd example/Qreport\_Sreport

$ mkdir run\_test

$ cd run\_test

$ ../../../bin/QReport -i ../test.fq.bz2 -l 51 -o my\_test\_output

and compare it with the provided run example, as specified in the README file under `./example/QReport_Sreport`

## Contributors

Paula Pérez Rubio

## License

GPL v3 (see LICENSE.txt)


---

Generated by  

 1.8.14
